# Knowledge-guided fuzzy logic modeling to infer cellular signaling networks from proteomic data

**DOI:** 10.1038/srep35652

**Published:** 2016-10-24

**Authors:** Hui Liu, Fan Zhang, Shital Kumar Mishra, Shuigeng Zhou, Jie Zheng

**Affiliations:** 1 Biomedical Informatics Lab, School of Computer Science and Engineering, Nanyang Technological University, Singapore 639798, Singapore; 2 Lab of Information Management, Changzhou University, Jiangsu, 213164 China; 3 Shanghai Key Lab of Intelligent Information Processing, School of Computer Science, Fudan University, Shanghai 200433, China; 4 Genome Institute of Singapore (GIS), A*STAR, Biopolis, Singapore 138672, Singapore

## Abstract

Modeling of signaling pathways is crucial for understanding and predicting cellular responses to drug treatments. However, canonical signaling pathways curated from literature are seldom context-specific and thus can hardly predict cell type-specific response to external perturbations; purely data-driven methods also have drawbacks such as limited biological interpretability. Therefore, hybrid methods that can integrate prior knowledge and real data for network inference are highly desirable. In this paper, we propose a knowledge-guided fuzzy logic network model to infer signaling pathways by exploiting both prior knowledge and time-series data. In particular, the dynamic time warping algorithm is employed to measure the goodness of fit between experimental and predicted data, so that our method can model temporally-ordered experimental observations. We evaluated the proposed method on a synthetic dataset and two real phosphoproteomic datasets. The experimental results demonstrate that our model can uncover drug-induced alterations in signaling pathways in cancer cells. Compared with existing hybrid models, our method can model feedback loops so that the dynamical mechanisms of signaling networks can be uncovered from time-series data. By calibrating generic models of signaling pathways against real data, our method supports precise predictions of context-specific anticancer drug effects, which is an important step towards precision medicine.

Signaling pathways are crucial for the cellular responses to environmental perturbations, via successive propagation of external stimuli to the downstream cellular components (e.g. transcriptional regulatory network)^1^, through which a cell can properly adjust its internal processes to adapt to the changing environment. A signaling pathway typically contains upstream proteins (e.g. receptors embedded in a cell’s membrane surface) that are responsible for sensing changes in the environment or directly involved in host-pathogen interactions. The upstream proteins can trigger a cascade of signal transduction events going through transmembrane receptors, intracellular signaling proteins and transcription factors, etc. Upon stimulation, the signal transduction may potentially lead to downstream alterations, such as activation or inhibition of chromatin remodellers and transcription factors (TFs), which consequently carry out regulatory programs of gene expression.

Many public databases, such as KEGG^2^ and Pathway Commons^3^, have been built to accommodate the increasing data and knowledge of signaling pathways. These databases, curated from literature and protein-protein interaction (PPI) networks, are instrumental for studying topological and structural characteristics of signaling networks, such as feedback loops, bistability and robustness against perturbations^4^,^5^. However, these canonical pathways are only represented as generic maps (signed and directed) consisting of the “routes” through which signal transduction is conducted. Also, these databases only provide cumulative evidence of signaling reactions involved in various types of cells and different perturbation conditions (e.g. drug treatments), which may not be applicable to a particular context. Since cellular responses to environmental cues are highly dynamic and context dependent, e.g., on cell types and exogenous environment, a crucial step in dynamical modeling of signaling pathways is to convert the generic maps to executable models using mathematical formalisms^6^,^7^ that can be calibrated against additional knowledge and real data. Such mathematical models can be used to simulate the dynamics of signal transduction *in silico*, through which we can save cost and time for wet-lab experiments and gain mechanistic insights into human diseases such as cancer.

Existing methods for modeling signaling pathways fall roughly into two categories: data-driven and knowledge-driven. Data-driven methods have emerged as popular tools for systems biology of signaling networks^8^,^9^. For example, Cai *et al*. proposed a Bayesian Network (BN) framework to integrate transcriptomic data with phosphoproteomic data to identify the pathways transmitting the signals of diverse stimuli in rat and human cells^10^. However, data-driven methods have a few limitations, such as the requirement of a large dataset for reliable inference and weak interpretability of molecular mechanisms. Knowledge-driven methods collect annotations of biochemical reactions from the literature, and encode them into executable models, typically in the form of Boolean networks^11^,^12^ or ordinary differential equations (ODEs)^13^,^14^. But current literature covers only parts of the genome-wide signaling network with limited mechanistic detail, and as such it is still impractical to build executable models for large-scale signaling networks with strong explanatory and predictive power.

Some phosphoproteomic datasets of cancer cells measured upon perturbations by chemical inhibitors, stimuli and knock-downs have been published^7^,^15^, which are valuable resources for uncovering real biochemical reactions and causal relationship among signaling proteins. However, the phosphoproteomic datasets available so far are mostly of small sizes and cover only a few stimuli and inhibitors, which restricts the applicability of data-driven methods. Therefore, some studies proposed hybrid approaches that combine the strengths of both knowledge-driven and data-driven methods. Specifically, using these methods we can manually curate signaling pathways from the literature and databases, use phosphoproteomic data to calibrate the network models and identify drug-induced rewiring of signaling pathways^16^,^17^,^18^,^19^,^20^. However, these existing hybrid methods do not explicitly model the feedback loops which are prevalent in signaling pathways. While linear regression-based hybrid methods^21^ have been developed for gene regulatory network inference, they are not directly applicable to the inference of signaling networks due to the sparse coverage of signaling proteins in the proteomic datasets available.

In this paper we propose a hybrid knowledge-guided network model based on fuzzy logic for signaling pathway inference. To make use of prior knowledge, two regularizers were introduced to impose penalties on violations of prior knowledge regarding individual edges and the maximum number of proteins directly interacting with each signaling protein. In particular, the dynamic time warping algorithm^22^ is adopted to measure the goodness of fit between experimental and predicted data, so that our method can model temporally-ordered experimental observations. Compared with existing methods of hybrid modeling, the contributions of our method lie in at least two aspects. First, a new knowledge-fused framework has been proposed which extends the *qualitative* informative priori proposed in^23^ to *quantitative* priori. Second, we have extended the traditional linear regression used in knowledge-fused methods to time series alignment so that our method can recover signaling pathways with feedback loops and uncover the dynamical mechanisms of signal transduction processes. Extensive empirical experiments have been conducted to analyze the performance improvement by different ratios of prior knowledge mixed with noise on a synthetic dataset. Also, the performance of the proposed model was evaluated on two real datasets, namely a dataset from the DREAM 4 Predictive Signaling Network Modeling Challenge^24^, and the phosphoproteomic and cell fate data of the apoptosis pathway with rewiring induced by sequential treatment of anticancer drugs^15^. The experimental results demonstrate that our model can uncover the signaling pathway alterations that are absent in the prior knowledge network but supported by independent perturbation data. Furthermore, it can successfully infer signaling pathways with feedback loops so that the dynamical mechanisms of cell fate determination can be uncovered from time-series data.

## Methods

**Figure 1:**
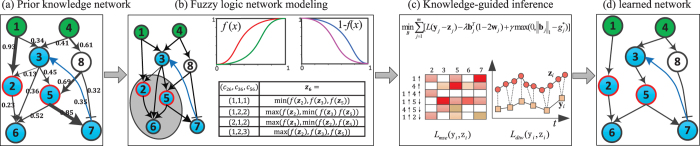
Schematic flowchart of our knowledge-guided network inference framework based on fuzzy logic modeling. (**a**) An illustrative signaling pathway with quantitative prior confidence scores assigned to individual edges. The stimulated proteins are colored in green, inhibited proteins are colored with red border, and the measured proteins are colored in cyan. A blue line represents a feedback loop. (**b**) Fuzzy logic modeling of signal transduction based on the normalized Hill function and the logical relation formulation among multiple input signals. (**c**) Knowledge-guild network inference based on fitting models to steady-state data by mean squared errors or time-series data by dynamic time warping. (**d**) The learned toy signaling network.

Our knowledge-guided fuzzy logic modeling for signaling pathway inference consists of four steps. As shown in Fig. 1, we present the flowchart of our method using a toy signaling pathway as an example.

### Building prior knowledge network

We first derive quantitative confidence scores from public knowledge bases of pathways to provide more accurate information than qualitative prior knowledge for the inference of the true network structure. The Pathway Commons database contains a large collection of annotations of biochemical reactions from various sources^3^, and each reaction is endorsed by a list of publications. The resource allows us to build a primary network which each edge can be assigned a quantitative confidence score. We adopt a scoring scheme similar to that proposed in ref. 25 to compute the prior confidence scores. In general, publications based on low-throughput techniques, due to a lower false positive rate, are considered to be more reliable than those based on high-throughput techniques, hence we assign higher confidence scores to low-throughput publications than high-throughput publications. First, each publication was classified as a high-throughput or low-throughput study according to the number of unique reactions supported by it. There are 56,193 publications and 4,228,150 reactions included in Pathway Commons, and the number of reactions endorsed in each of 90% publications is less than 111. Therefore, a publication associated with more than 111 reactions would be classified as high-throughput and otherwise low-throughput. Next, if a reaction is endorsed by only one type of study (either high- or low-throughput but not both) we assign confidence score according to Table 1. If a reaction is endorsed by both high- and low-throughput studies, the confidence score is equal to the confidence score of the low-throughput column in Table 1 plus 0.05 times the number of high-throughput endorsements. If the computed value of the confidence score exceeds 0.95, it is still assigned a maximum value of 0.95.

**Figure 2:**
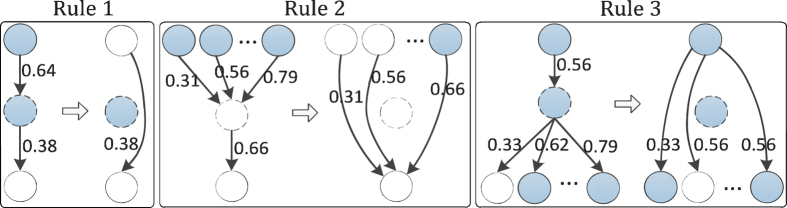
Three rules used to compress a primary signaling network and update the prior confidence scores of edges. The nodes drawn in dashed lines represent proteins removed in the compression procedure, and the nodes drawn in solid lines represent reserved proteins.

From the primary network with quantitative confidence scores, we extract a subnetwork that covers as completely as possible the receptors of interest, perturbed and measured proteins included in a phosphoproteomic dataset. However, the primary subnetwork may contain some non-identifiable elements that should be removed so that the network calibration against real data can be more efficient. For clarity, we define the manipulated or experimentally measured proteins as “designated” nodes, and other proteins as “undesignated” nodes. We extend the three rules proposed in^16^ to compress the primary networks and adjust the confidence scores simultaneously: 1) A signaling cascade consisting of a series of “undesignated” proteins and reactions directed to a “designated” node should be compressed, while all “designated” nodes are reserved in the process of compression; 2) a linear cascade converging to dispatcher-like proteins should be compressed; 3) multiple outgoing signals diverging from a linear cascade in which no protein is manipulated or measured should be compressed. Whenever a node is removed and two tandemly associated edges are merged into one new edge, the smaller of the confidence scores associated with the two original tandem edges would be reserved as the confidence score of the new edge, because the edge with a lower score is the bottleneck of a pathway through which some flux can go. Figure 2 illustrates an example for each compressing rule, as well as the adjustment of confidence scores.

### Fuzzy logic network modeling

To generate simulation data from the prior knowledge network, we adopt a fuzzy logic model to describe the effects of input signals on each node (activations or inhibitions). Compared with Boolean logic models, fuzzy logic models can describe the gradual responses and typical sigmoidal biological reactions that can not be modeled by Boolean networks. Here, we adopt the normalized Hill function^26^ to transform the input signals to the output signal. Let a real-valued variable 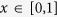 denote a single input signal, then its activation effect can be formulated as


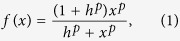


where *p* (>0) is the Hill coefficient which is an integer and determines the steepness of the sigmoidal transition between the activation and inhibition states, and *h* (>0) is t*h*e sensitivity parameter which is a real number and determines the midpoint of the Hill function. Note that the value of *f(x*) falls in the range of [0, 1], and thus the inhibition effect is accordingly defined as 1−*f(x*). In the case that multiple inputs exist, the AND gate is used if all inputs must be active to activate the output node, and the OR gate is used when the output node would be activated as long as at least one input node is active. In particular, we give the AND gates a higher priority than the OR gates among multiple logical gates in a logical expression. This priority order corresponds to the disjunctive normal forms (DNF), i.e. Boolean variables or negated Boolean variables comprise a few AND clauses, and the AND clauses are in turn connected by OR operations. In fact, any particular Boolean function can be represented by one and only one disjunctive normal form^27^. Moreover, to extend the DNF to a fuzzy logic model, AND and OR gates are usually substituted by *min* and *max* operators so that continuous values can be handled.

Given the Hill function and logical operations mentioned above, our goal is to infer the actual logical relationships from the real data. However, the number of possible logical combinations of *n* input signals is a super-exponential function (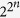), hence it is computationally inhibitive to enumerate all possible logical combinations for even a moderate-sized network. In a specific cellular context, this problem can be simplified by assuming that a regulator can activate or inhibit a target protein alone or by synergizing with a specific set of co-regulators, although in a different cellular context this regulator may collaborate with other proteins. As such, among all possible combinations of multiple regulators, we focus on a subset of logical forms that cover a vast majority of biologically realistic cases. In terms of DNF, this assumption means that a Boolean variable would appear in one and only one AND clause regarding a certain objective variable. As a result, we propose a coding scheme that can represent all such logical combinations narrowed down by the assumption from all possible DNFs.

Formally, a matrix of binary variables ***R*** = (**r**_1_, …, **r**_*m*_) is used to represent the regulation type (activations or inhibition), in which the element *r*_*ij*_ is equal to 1 if the *i*-th node activates the *j*-th node, and 0 if the regulation type is inhibition. Another matrix ***C*** = (**c**_1_, …, **c**_*m*_) is introduced where the *j*-th column **c**_*j*_ = (*c*_1*j*_, …, *c*_*mj*_)^*T*^ is an integer vector encoding the logical relationships among the input signals for the *j*-th node which is explained in the following. Assume that the value of *c*_*ij*_ corresponding to the edge from node *i* to node *j* belongs to {1, 2, …, ||**b**_*j*_||_1_}, then for another incoming edge from say node *k* to node *j* (i.e. *b*_*kj*_ = 1 and *k* ≠ *i*), we check whether *c*_*kj*_ is equal to *c*_*ij*_. If *c*_*kj*_ = *c*_*ij*_, then the AND logical relation is assigned between the two input signals. Note that ||**b**_*j*_||_1_ is equal to the number of input signals of node *j*. Hence, by iteratively assigning each element in **c**_*j*_ to an integer value from 1 to ||**b**_*j*_||_1_, we can enumerate all AND clauses. Thereafter, all the AND clauses are linked by OR operations to form the final logical relation. Mathematically, let 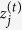 be the state of node *j* on the *t*-th iteration, and we update its state by calculating the following logical expressions based on the states of its parent nodes on (*t*−1)-th iteration:


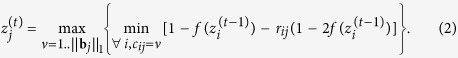


Note that the term in the square bracket is equal to 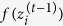 if *r*_*ij*_ = 1 (activation effect), and equal to 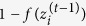 if *r*_*ij*_ = 0 (inhibition effect), respectively. As an illustration, we present a few examples of the logical expressions regarding the 6-th node in the toy pathway, as shown in Fig. 1(b).

### Knowledge-guided network inference

In this section, we present a method to calibrate the prior knowledge network by fitting simulated data generated by the fuzzy logic model with real data, thereby inferring context-specific signaling network models. A signaling network is often represented as a directed graph *G* = (*V, E*), where the set of vertices (or nodes) *V* represents a collection of *m* signaling molecules, and the set of edges *E* represents the biochemical reactions between the signaling molecules. Let 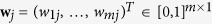 be the confidence scores of the edges incident on the *j*-th node (*j* = 1, 2, …, *m* hereafter). Let *B* = (**b**_1_, …**b***m*) be the adjacency matrix of the graph to be inferred, in which the *j*-th column **b**_*j*_ = (*b*_1_, …, *b*_*mj*_)^*T*^ is a binary vector encoding whether a node is a parent of the *j*-th node. Assuming 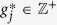 represents the knowledge-derived threshold of the maximum number of incoming edges incident on the *j*-th node, our knowledge-guided network inference is formulated as the following optimization problem:


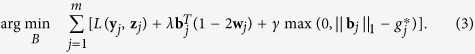


*L*(**y**_*j*_, **z**_*j*_) is a non-negative real-valued *loss function* that measures the difference between experimental data 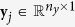 and predicted data 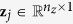, where *n*_*y*_ and *n*_*z*_ are the numbers of experimental and predicted samples, respectively. The latter two terms, namely 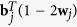 and 
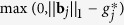
, where 
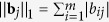
 is the number of incoming edges (i.e. indegree) of the *j*-th node, are regularizers that impose penalties on violations of prior knowledge and network complexity. The two parameters *γ* and *λ* are both non-negative real variables introduced to balance the goodness of fit, violations of prior knowledge and model sparsity.

Mukherjeea and Speed^23^ proposed a qualitative informative prior:


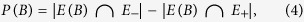


in which *E*_ + _is the set of prior edges and *E*_−_ is the complement edge set of *E*_ + _. Note that the second term 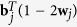 can be rewritten as 
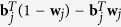
, which essentially corresponds to Equation (4) and extends the qualitative prior to quantitative prior. The third term 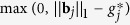 imposes a penalty on the *j*-th node if and only if the number of its incoming edges exceeds the knowledge-derived threshold 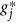. The underlying rationale is that, although the number of regulators of a signaling molecule may be big in a prior knowledge network, the signaling molecule is regulated by a very small number of regulators in a specific cellular context. In fact, limiting the number of incoming edges has been shown to help yield biologically more realistic network models^28^,^29^. The free parameters *γ* and *λ* represent the weights of the two regularizers. Suppose that we hold equal confidence in the two types of priori, *γ* can be set equal to *λ* and thus the number of free parameters is reduced from two to one.

The calculation of the loss function depends on whether the experimental data are steady-state or dynamical time-series data. If each experimental observation is obtained when a steady state is reached upon a specific perturbation, predictions are made by simulating the virtual perturbations on the candidate signaling network and running the dynamic simulation till a steady state is reached. In such a scenario, the loss function can be formulated as the *mean squared error* (MSE) between the experimental and predicted data:


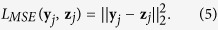


In some cases, the experimental data consist of a series of temporally-ordered measures of the activity levels of signaling molecules, e.g. experimental observations at 8 discrete time points within 48 hours after the exposure of breast cancer cells to combinatorial drugs^15^. Accordingly, we can simulate the dynamic cellular responses to drug perturbations on the candidate signaling network, whereby time series are generated to describe the dynamic activity levels of signaling molecules. Since the simulated time-series data can be much denser than the experimental data, i.e. *n*_*z*_ > *n*_*y*_, the traditional mean squared error loss function is not applicable. Therefore, we exploit the temporal alignment method, *dynamic time warping* (DTW)^22^, to measure the divergence between the experimental and predicted time series, as illustrated in Fig. 1(c). Formally, let *S* be the number of steps needed to align the two time series, and let 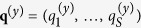 and 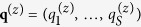 be the correspondence indexes representing the aligned compositions between **y**_*j*_ and **z**_*j*_, then the loss function is defined as


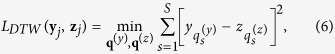


which is subjected to the constraints of continuity (
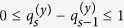
; 
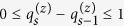
) and monotonicity (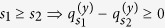 and 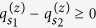). The dynamic time warping problem can be efficiently solved by using the dynamic programming algorithm to find the optimal correspondence vectors in linear time and space^30^.

To simulate data from a candidate network, the fuzzy logic model described in Section “fuzzy logic network modeling” is used to compute the state of each node, and generate simulation data for given input signals. According to the type of experimental data, we employ two different configurations to simulate steady-state or temporally-ordered data respectively. For steady-state experimental data, the values of the receptor nodes of each sample is taken as input and we run the simulation till a stable state is reached or a predefined maximum iterations is exceeded, and thus generate equal number of simulation data points as the experimental data. The stable state is judged by checking whether the absolute difference of two successive simulated states is less then a small threshold *ε*. In our implementation, *ε* is set to 1.0e-6, and the maximum iterations is heuristically set to 5 times of the number of network nodes.

For temporally-ordered experimental data, simulation data can be generated by successively choosing some nodes to update their states and repeating the update process. In particular, two modes, synchronous and asynchronous, can be used to simulate data from a candidate network. For synchronous mode, the states of all nodes are simultaneously updated at each time point, and the successively generated values constitute the simulated time-series data. For asynchronous mode, only one node are randomly chosen to update its state at each time point. Since asynchronous mode is computationally inhibitive for even moderate-sized networks, we employed the synchronous mode in our implementation. Due to the fact that feedback loops contained in a network would lead the simulation to oscillations, i.e. we can never reach a stationary stable state. Therefore, we run the simulation to generate time-series data and stop the simulation process when the number of time points is three times the experimental number of time points in the real data.

Our main task is to infer the values in the matrices *B, C* and *R* from the experimental data. Since all the variables to be inferred are integers, we reformulated the minimization problem defined in Equation (3) into a constrained nonlinear integer programming (NLIP) problem, which is formally defined as follows:


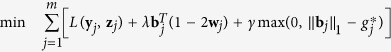


*s*.*t*.


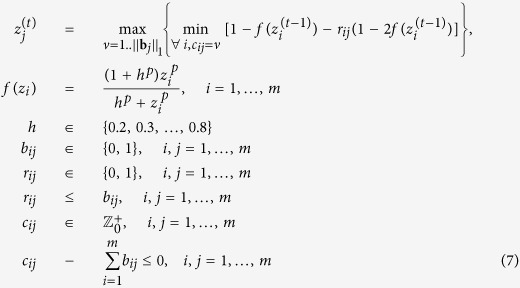


in which 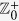 represents the set of non-negative integers. We utilize the genetic algorithm (GA) included in the MATLAB optimization toolbox (MATLAB R2012b release) to solve the NLIP problem, considering that the GA is often able to find the approximate global optimum in most cases. We ran the algorithm 50 times so that the global or nearly global minimum is likely to be found, and the optimum was chosen as the final result. For the Hill coefficient *p* and activation coefficient *h* in Equation (1), we empirically set their values using the grid-like search strategy that have been used in previous studies^26^,^31^. As a result, we empirically set *p* to 2, and specified *h* to 0.2, 0.3, …, or 0.8. Such configurations can encode a large number of different models to fit to the real data. After testing the models with other values of *p* and *h*, which allows us to infer the parameters in a larger solution space, we did not find that additional configurations could significantly improve the goodness of fit to the real data than the aforementioned subset of the specified values.

### Data Availability

The source code and datasets are available at the GitHub site: https://github.com/hliu2016/fuzzy.

## Results

### Empirical analysis of synthetic data

**Figure 3:**
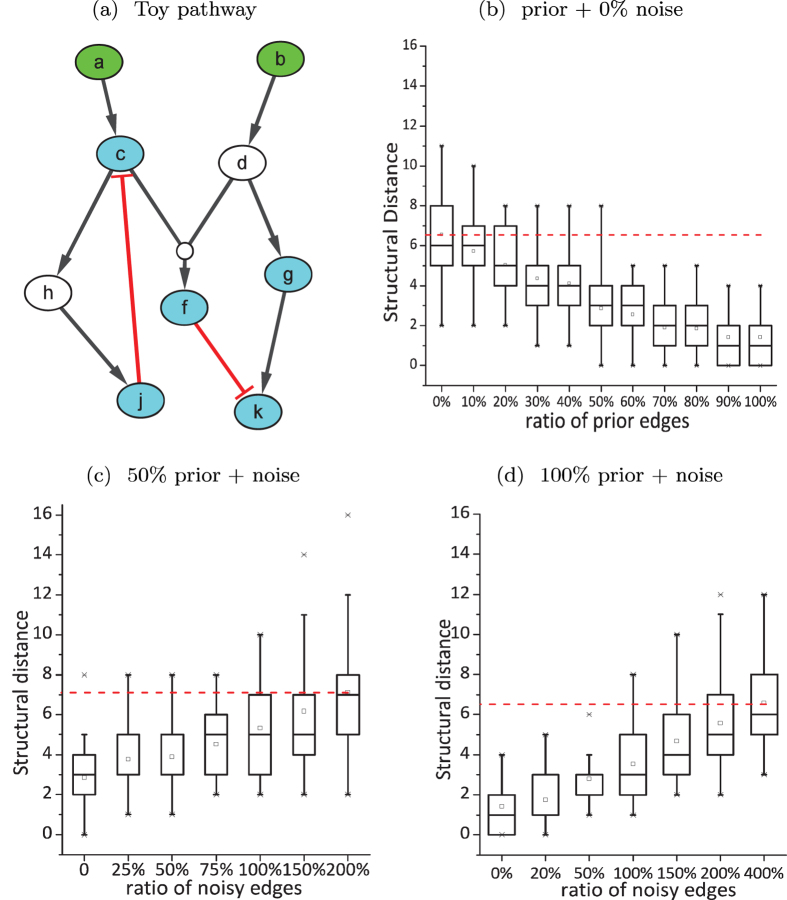
A case study of performance of network inference on synthetic data under different ratios of prior and noisy edges. (**a**) The signaling pathway used to generate the synthetic data. The stimulated proteins are colored in green, and measured proteins are colored in cyan. The small circle merging edges from nodes *c* and *d* into the edge coming to node *f* represents an AND logical gate; (**b**) The boxplot of structural distance (SD) under different ratios of prior edges without noisy edge; (**c**) The boxplot of SD with 50% prior edges and different ratios of noisy edges; (**d**) The boxplot of SD with 100% prior edges and different ratios of noisy edges.

To evaluate the contribution of prior knowledge to the inference of signaling pathways, we analyzed synthetic data to estimate the effect of varying ratios of the number of prior edges to the total number of edges in a network, as well as different ratios of noisy edges, on the reverse engineering of network structures. The signaling pathway used to generate the synthetic data is shown in Fig. 3(a). It comprises 9 nodes and 10 edges, and there is a negative feedback loop *c* → *h* → *j* ⊣* c*. We used the R package of BoolNet^32^, which is an implementation of Boolean network, to produce 5 time-series datasets. Each time-series dataset includes 10 successive states along a trajectory from a random initial state. To mimic the discrete experimental observations, we randomly extracted 6 or 7 states (the initial state was included) from each time-series dataset to build the synthetic data. For each time series, we predicted 10 successive states from the initial state based on a candidate network and then used the dynamic time warping algorithmto align the predicted time series with the synthetic time series.

#### Prior edges significantly improve performance

We first investigated whether the prior knowledge could improve the performance of our model in predicting time-series data. For this purpose, we used varying ratios (0–100%) of true edges (i.e., prior edges) as prior knowledge to guide the model inference. The ratio of the prior edges is defined as 
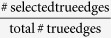
. For each ratio, the prior edges are randomly selected from the true network topology, and they are assigned confidence scores randomly generated by drawing from a uniform distribution between 0 and 1. The maximum number of incoming edges for each node was set to 2, and the parameters *γ* and *λ* were both set to 0.5. We used the structural distance (SD) between the learned and the true networks, where the SD is defined as the total number of additions and deletions of edges needed to convert the learned network into the true network. As such, smaller values of SD indicate better performance in recovering the true network structure. To avoid potential bias in selecting the prior edges, we repeated the procedure 20 times for each specific ratio. Figure 3(b) shows the boxplot of SD with respect to varying prior ratios. We found that the accuracy was significantly improved when the ratio of prior edges increased. For example, as shown in Fig. 3(b), the mean of SD at 50% prior is significantly less than that at 0% prior (*t*-test, *p*-value ≤ 0.0052). These results demonstrate that prior knowledge can significantly improve the performance of signaling pathway inference.

#### Noise hinders performance improvement by prior knowledge

**Figure 4:**
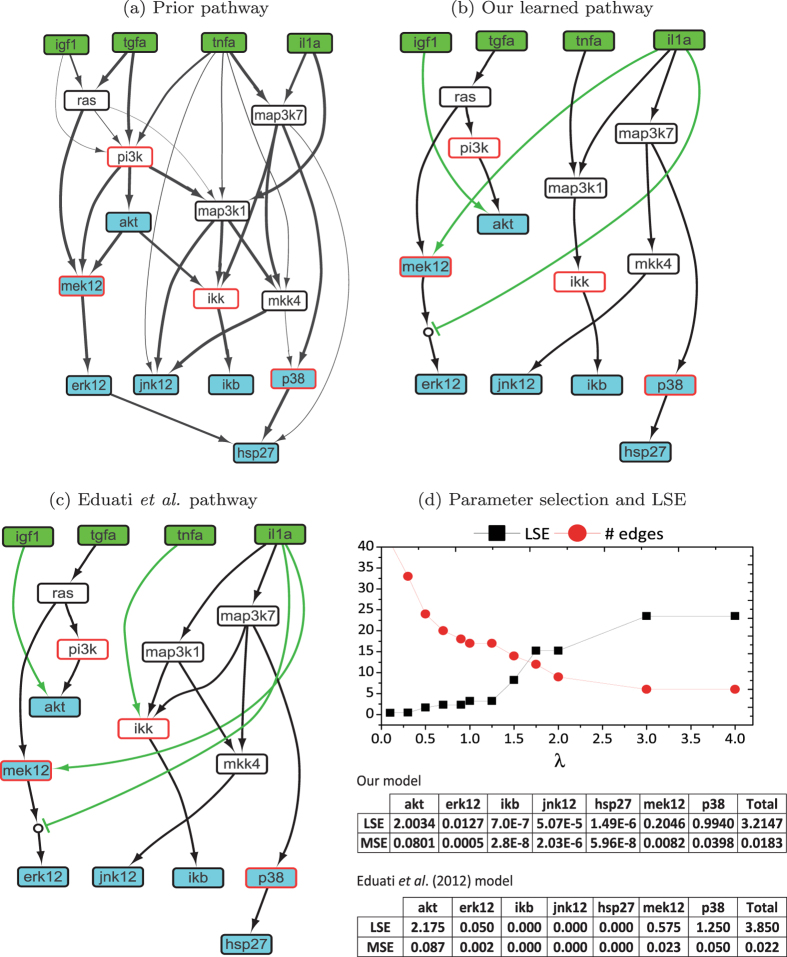
Comparisons among the prior pathway and two learned pathways. (**a**) The prior knowledge pathway was derived by removing unidentifiable proteins in the original network published for the DREAM 4 challenge^24^. The width of each edge is proportional to its prior confidence score. (**b**) The signaling pathway learned by our method in which the green edges were inferred and added into the prior network. (**c**) The signaling pathway learned in ref. 19 in which the green edges were inferred although not in the prior network. (**d**) The curves of the best LSE and model complexity (measured by the number of edges) with regard to *γ*, as well as the LSE and MSE values for the measured proteins.

To assess the robustness of the contribution of prior knowledge to the performance improvement, we introduced different ratios of false positive edges (i.e., noisy edges). The ratio of noisy edges is defined as 
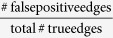
. The confidence scores for both true and false positive edges were randomly drawn from the uniform distribution between 0 and 1. Similarly, the procedure was repeated 20 times to avoid bias in edge selection. As shown in Fig. 3(c,d), the mean of SD increases gradually with the ratio of noisy edges, indicating that noisy edges can hinder the performance improvement by prior knowledge. Note that, with the ratios of noisy edges near 1.5 in Figs 3(c) and 4 in Fig. 3(d), the mean SD values (≈6) corresponding to 50% prior and 100% prior edges respectively are approximately equal to that learned without any prior information or noise (i.e., 0% prior edges and 0% noise), as shown by the red dashed line in Fig. 3(b–d). This observation suggests that the prior knowledge would be helpful in uncovering the true network unless multifold noisy edges are introduced into the prior knowledge, indicating the robustness of the performance improvement by using prior knowledge.

### Evaluation on steady-state data

A phosphoproteomic dataset of human liver cell line (HepG2) was used for the DREAM 4 Predictive Signaling Network Modeling Challenge^24^. Based on the dataset, we conducted performance comparison of our method with existing data-driven and hybrid methods. The phosphoproteomic dataset includes 25 samples measured in steady states, and each phosphorylation level was normalized to the range (0, 1) by the logistic transformation proposed in^16^. From the original signaling pathway provided by the DREAM 4 organizers, we derived a compressed network as shown in Fig. 4(a), in which the stimulated nodes are colored in green, the inhibited nodes in red border line and the measured nodes in cyan. We calculated the confidence score for each edge of the simplified network by using the method described in Section “Building prior knowledge network”. As shown in Fig. 4(a), the width of each edge is proportional to its confidence score, and the maximum number of incoming edges for each node was set to 3.

#### Performance comparison

As data-driven methods can only infer the links limited to perturbed and measured proteins which constitute only a subset of the proteins of interest, the resulting networks often have weak biological interpretability. For example, we ran the Bayesian network algorithm MMHC^33^ and markov blanket algorithm HITON_PC^34^ included in the MATLAB toolbox Causal Explorer^35^ to infer the signaling pathway, and found that the resulting networks were difficult to interpret, as they include edges between perturbed and measured proteins and are very different from the original signaling pathway provided by the DREAM 4. Therefore, we focused on the comparison with another hybrid method proposed in ref. 19, which was built on the method with the best performance out of twelve groups participating in the DREAM 4 challenge^8^. Given the prior network with confidence scores as input, we trained our model to fit the experimental data, and the parameters *λ* and *γ* were empirically set to 1.2, which achieved a good balance between the LSE (least square error) and the penalty term in Equation (3). The resulting network contains 16 edges, as shown in Fig. 4(b). Moreover, the LSE of each measured protein is presented in Fig. 4(d). Based on the prior network, three edges (IGF1 → AKT, PI3K → ERK12 and IL1a → MEK12) were added to our learned network, and an AND logic gate (MEK12 ^ ¬ IL1a → ERK12) was derived. Compared with the network in Fig. 4(c) as learned in ref. 19, our method achieved better network sparsity and goodness of fit to the real data, which were the two main measures for performance evaluation in the DREAM 4 challenge. For clarity, we converted the LSE into MSE values as defined in ref. 19 using the formula MSE = LSE/25 and the total MSE is equal to Σ_*i*_ LSE/7. Note that the total MSE value obtained by our method is less than that reported in ref. 19 (0.0183 *vs.* 0.022). Moreover, our model contains totally 17 edges including 3 added edges (in green color), while the model in ref. 19 consists of totally 19 edges including 4 added edges, suggesting that our model has lower complexity without compromising the goodness of fit to the real data.

#### Statistical significance of learned model

To test if the learned model is statistically significant given the experimental data and prior network, we generated a set of random networks. Each random network was generated according to the following rules: 1) it should have the same numbers of nodes and edges as the prior network; 2) each node is incident to at least one edge and each stimulated node has no incoming edge but at least one outgoing edge; 3) the confidence scores are randomly selected from the original prior scores. The randomly generated networks are taken as the “random prior knowledge” to guide the models, and the procedure is repeated 500 times. We found that the random prior networks yielded significantly larger LSE values than the true prior network (*z*-score = 3.5675, *p*-value < 0.000183). The results indicate that randomly generated models are unlikely to fit the experimental data with comparable goodness of fit as the knowledge-guided model.

### Evaluation on single-cell time-series data

It has been demonstrated that the triple-negative breast cancer cell line (BT-20) is sensitive to sequential and time-dependent combinatorial drug treatments using the EGFR inhibitor Erlotinib and DNA damage inducer Doxorubicin^15^. It was observed that the BT-20 cells did not show significantly apoptotic response when treated by (1) either drug alone, or (2) both at the same time, or (3) Erlotinib shortly before Doxorubicin. Strikingly, if Erlotinib was added at least 4 hours, especially 24 hours, prior to adding Doxorubicin (denoted as E → D treatment), the cancer cells showed as much as 500% increase in cell death compared to the reverse order of drug usage or using Erlotinib alone. The sequential and time-dependent drug treatment is supposed to rewire the signaling pathways and trigger the signaling cascade of apoptosis.

**Figure 5:**
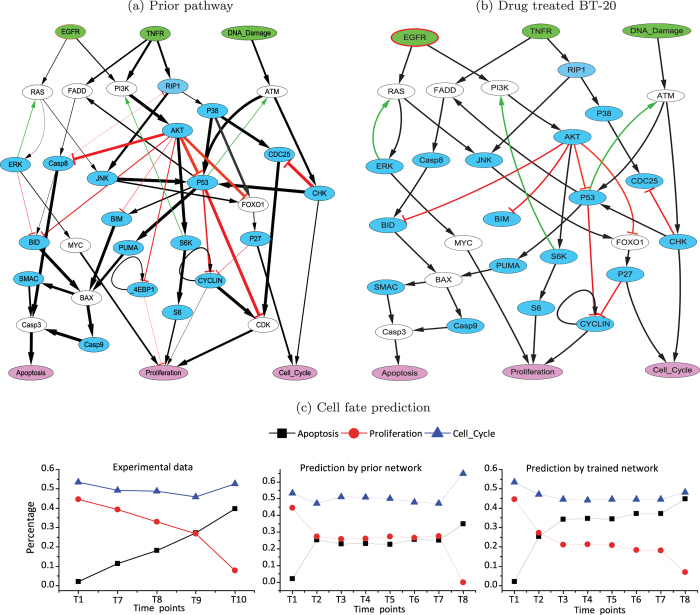
Predictions of cell fates by prior and learned networks. (**a**) Prior signaling network derived from two manually curated signaling pathways proposed in our previous studies^36^,^37^. The black and red edges represent interactions of types activation and inhibition, and green edges represent feedback interactions. (**b**) Network inferred by using the phosphoproteomic time-series data of the E → D treated BT-20 cell line. (**c**) Dynamic curves of three cell fates, i.e. apoptosis, proliferation and cell cycle arrest, corresponding to experimental observations, the predictions by the prior network and trained network, respectively.

Therefore, we used our method to detect the alterations of signaling pathways induced by the combinatorial drugs. As shown in Fig. 5(a), the model of the prior network comprises mostly the signaling proteins involved in apoptosis and DNA damage response. In particular, the “DNA damage” node represents the DNA damage signal induced by Doxorubicin. The three pink nodes labeled with “Apoptosis”, “Proliferation” and “Cell cycle” represent the cell fates that can be attributed to the activities of the signaling pathways. The single-cell phosphoproteomic dataset published in^15^, which is used here for model calibration, consists of the phosphorylation levels of 20 signaling proteins (cyan nodes) over 8 discrete time points. Moreover, the cell counting corresponding to the three types of cell fates was measured by flow cytometry, which allows to evaluate our method by checking whether the inferred signaling network model can capture the dynamic trends of cell fates induced by the drug treatment.

First, we used the single-cell time-series data of the BT-20 cell line upon the E → D treatment to calibrate the prior network. In particular, we ran the simulations using the fuzzy logic network model (as described in the Methods section) with the synchrnonous updating scheme till a steady state was reached or a predefined maximum number of iterations was exceeded. To conduct the temporal alignment, a sliding window moving along the simulated continuous time-series data was used to extract a fragment of data, which was taken as input by the aforementioned dynamic time warping (DTW) algorithm to align with the experimental data. The size of the sliding window was set to three times the experimental data, i.e. 3 × 8 = 24 time points. The learned network is shown in Fig. 5(b), which contains less edges than the prior network. It is worth noting that the cell fate data were not used in the learning stage, so that the model evaluation in terms of cell fate prediction would be more objective than direct comparisons of phosphorylation levels. For prediction and validation, however, the three cell fate nodes were added into the learned network by linking to them from the upstream signaling molecules according to the prior network. Secondly, we ran simulations to arrive at steady states and then counted the number of truths for each type of cell fate. Because the cell fate nodes had not been included in the fuzzy logic network model for inference, we did not have the parameter values for the edges linking signaling nodes to the cell fate nodes. To reduce the dependency on parameters, we converted the fuzzy logic model to Boolean network for the prediction of cell fates. To this end, we binarized the phosphoproteomic data of the 20 measured signaling proteins as their initial states, and generated random Boolean initial states for other proteins. Then, the initial state was taken to simulate the dynamics of both the prior and trained networks using the BoolNet software^32^. Note that, for dynamic simulations, our learned fuzzy logic network can function as a Boolean network when given Boolean inputs. Corresponding to each of the 8 time points, we repeated the procedure for 10,000 times and recorded the number of truths in steady states for each type of cell fate. Finally, we computed the percentage of each type of cell fate at each time point, and plotted the curves connecting the dots corresponding to the percentages to describe the dynamic changes of cancer cell fates.

As shown in Fig. 5(c), the experimental observations suggested that the percentage of cell death is increasingly induced and proliferation was significantly inhibited upon the E → D drug treatment. Compared to the prior network, the trained network performed better in capturing the dynamic trends of the cell fate transitions. Note that the flow cytometry data of cell fate measurements comprise 12 time points, i.e. 0 h, 0.1 h, 0.25 h, 0.5 h, 1 h, 1.5 h, 2 h, 4 h, 6 h, 8 h, 12 h and 24 h. For clarity, we denote the time points by T1, T2, ..., T12, respectively. The experimentally observed cell fates comprise only 5 time points (T1, T7, T8, T9 and T10), while the phosphoproteomic data consist of 8 time points (T1, T2, T3, T4, T5, T6, T7, T8). The predicted cell fates are based on the phosphoproteomic data and thus comprise 8 time points, as shown in Fig. 5(c). Considering potential time delays between the drug-induced changes of signaling pathways and experimentally observed changes of cell fates, we excluded the middle three time points (T2, T3 and T4) of the predicted data and then aligned the rest time points to the experimental data. As a result, we can calculate the correlation coefficients between the experimentally observed cell fates and predicted cell fates. For apoptosis and proliferation, the Pearson’s correlation coefficients were 0.8968 *vs.* 0.7292 and 0.8698 *vs.* 0.7902 for the trained network *vs.* prior network, respectively. For cell cycle, the trained network and prior network achieved about equal Pearson’s correlation coefficients of 0.6352 and 0.6186. The results indicate that our method effectively calibrated the prior network model by using the phosphoproteomic time-series data, thereby making more accurate predictions of cancer cell fates.

## Conclusion and Discussion

Although purely data-driven methods have achieved remarkable success in modeling biological networks, the results are often difficult to interpret in terms of molecular mechanisms. With the accumulation of domain knowledge, we expect to integrate the knowledge and experimental data to produce executable mathematical models, and thereby uncover hidden mechanisms of cellular phenotypes. We have high confidence in the domain knowledge and expect that the models calibrated against real data would not diverge too much from the knowledge-based model. In particular, we extended the qualitative informative priori proposed by Mukherjeea and Speed^23^ to quantitative priori. In many situations, our confidence in prior knowledge is not entirely definite (e.g. true or false), but often uncertain. For instance, literature-derived evidence for different phosphorylation reactions in a specific cancer cells are often not equally strong, i.e. some reactions are strongly supported while other are only moderately supported by previous findings. Therefore, real-valued fuzzy confidence scores (ranging from 0 to 1) of prior knowledge possesses more descriptive power than binary prior information.

To the best of our knowledge, we are the first to introduce the time-series alignment technique into the signaling pathway inference. As such, our method can handle not only steady-state experimental data, but also temporally-ordered experimental data, whereas previous studies can only address steady-state experimental data^8^,^26^,^31^. Furthermore, the capacity of handling time-series data allows us to recover signaling pathways with feedback loops and reveal dynamical mechanisms of signal transduction. It is known that feedback loops in a network often lead to oscillatory states which is beyond the capacity of previous methods that depend on linear regression of steady-state data.

**Figure 6:**
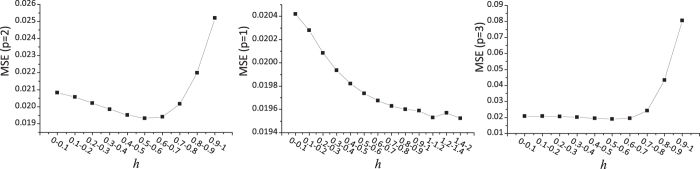
The impacts of the parameters *h* and *p* in the normalized Hill function on MSE. For each value of *p* (1, 2 or 3) and *h* falling into each of the 10 equal-length intervals, the optimal MSE is obtained by running the genetic algorithm for 50 times.

As fuzzy models can describe the gradual responses and typical sigmoidal biological reactions, we adopted the fuzzy model rather than Boolean model. The normalized Hill function is widely used in kinetic modeling of biochemical reaction networks and its two parameters *p* and h are simply determined by a grid-like search used in previous studies^26^,^31^. To verify the validity of the search strategy, we explored the impact of *p* and *h* on the MSE, using the DREAM 4 dataset (for more details see subsection titled “Evaluation on steady-state data”). To capture typical sigmoidal biological reactions, the value of *p* should be greater than 1, but a large value of *p* would lead to a steep curve representing the asymptotic trend. Therefore, we set the value of *p* to 1, 2, and 3 respectively, and divided the range of [0, 1] for *h* into 10 equal-length intervals, i.e. [0, 0.1], [0.1, 0.2], …, [0.9, 1]. For each pair of values of (*p, h*), the optimal MSE was obtained by running the genetic algorithm for 50 times. As shown in Fig. 6, the MSE decreases gradually with the increase of *h*, but the MSE rises rapidly when *h* is greater than 0.8 except for the linear transfer (*p* = 1). Hence, we specified the value of *h* to 0.2, 0.3,…, 0.8, which can be optimized along with other variables by the genetic algorithm. To capture typical sigmoidal biological reactions, *p* was set to 2. Besides, it looks that the magnitudes of impacts on MSE of the two parameter *p* and *h* are much less than the model parameter *λ*, as shown in Fig. 4(d). Therefore, we focus on adjusting the value of *λ* to balance the MSE and the penalty on the violation of prior knowledge in our empirical experiments.

An important objective of our paper is to construct cellular context-specific network models for cancer cell fate decisions, especially apoptosis induced by drug treatments. The hybrid model proposed in this paper is an important step toward this goal, by calibrating generic models of signaling pathways against quantitative phosphoproteomic data measured in cancer cells. However, in real cancer cells the rewiring of signaling pathways must undergo the transcriptional programs and metabolic processes before the induced cell fates can be observed, i.e., there is a time delay between the drug-induced changes of signaling pathways and experimentally observed changes of cell fates. Since the predicted cell fates in this paper were directly based on the signaling networks, there is a sharp rise of the proportion of dead cells, as shown in Fig. 5(c), whereas the experimentally observed proportions of cells committing suicide (e.g. apoptosis) change gradually and smoothly, probably due to the time lags from the signal transduction to phenotypic changes. Therefore, we plan to link the signaling pathways and transcriptional regulatory networks, thereby taking the time delays into account. Moreover, we will incorporate genomic and transcriptomic data of individual patients, such as somatic mutations, copy number variations and gene expressions data, to further calibrate the models, so that it can precisely predict the context-specific anti-cancer drug effects, thereby help pave the way for the precision medicine.

## Additional Information

**How to cite this article**: Liu, H. *et al*. Knowledge-guided fuzzy logic modeling to infer cellular signaling networks from proteomic data. *Sci. Rep.* **6**, 35652; doi: 10.1038/srep35652 (2016).

## Figures and Tables

**Table 1:** Confidence score assignments according to low-throughput or high-throughput studies.

Number of endorsed studies	Low-throughput	High-throughput
0	0	0
1	0.8	0.25
2	0.9	0.5
3	0.95	0.75
≥4	0.95	0.85

## References

[b1] Jorgensen C. & Linding R. Simplistic pathways or complex networks. Curr Opin Genet Dev 20, 15–22 (2010). 20096559 10.1016/j.gde.2009.12.003

[b2] Kanehisaa M. & Goto S. KEGG: kyoto encyclopedia of genes and genomes. Nucleic Acids Res 28, 27–30 (2000). 10592173 10.1093/nar/28.1.27 PMC102409

[b3] Cerami E. . Pathway Commons, a web resource for biological pathway data. Nucleic Acids Res 39, D685–D690 (2011). 21071392 10.1093/nar/gkq1039 PMC3013659

[b4] Calzone L. . Mathematical modelling of cell-fate decision in response to death receptor engagement. PLoS Computational Biology 6, e1000702 (2010). 20221256 10.1371/journal.pcbi.1000702 PMC2832675

[b5] Legewie S., Bluthgen N. & Herzel H. Mathematical modeling identifies inhibitors of apoptosis as mediators of positive feedback and bistability. PLoS Computational Biology 2, e120 (2006). 16978046 10.1371/journal.pcbi.0020120 PMC1570177

[b6] Fisher J. & Henzinger T. A. Executable Cell Biology. Nat Biotechnol 25(11), 1239–1249 (2007). 17989686 10.1038/nbt1356

[b7] Samaga R., Saez-Rodriguez J., Alexopoulos L. G., Sorger P. K. & Klamt S. The logic of EGFR/ErbB signaling: theoretical properties and analysis of high-throughput data. PLoS Computational Biology 5, e1000438 (2009). 19662154 10.1371/journal.pcbi.1000438 PMC2710522

[b8] Eduati F., Corradin A., Di Camillo B. & Toffolo G. A Boolean approach to linear prediction for signaling network modeling. PloS One 5 (2010). 10.1371/journal.pone.0012789 PMC2940821 20862273

[b9] Janes K. A. & Yaffe M. B. Data-driven modelling of signal-transduction networks. Nature Reviews Molecular Cell Biology 7, 820–828 (2006). 17057752 10.1038/nrm2041

[b10] Cai C., Chen L., Jiang X. & Lu X. Modeling signal transduction from protein phosphorylation to gene expression. Cancer Inform 13 (Suppl 1), 59–67 (2014). 10.4137/CIN.S13883 PMC4216050 25392684

[b11] Albert I., Thakar J., Li S., Zhang R. & Albert R. Boolean network simulations for life scientists. Source Code for Biology and Medicine 3, 16 (2008). 19014577 10.1186/1751-0473-3-16 PMC2603008

[b12] Mai Z. & Liu H. Boolean network-based analysis of the apoptosis network: irreversible apoptosis and stable surviving. Journal of Theoretical Biology 259, 760–769 (2009). 19422837 10.1016/j.jtbi.2009.04.024

[b13] Hughey J., Lee T. & Covert M. Computational modeling of mammalian signaling networks. Wiley Interdisciplinary Reviews: Systems Biology and Medicine 2, 194–209 (2010). 20836022 10.1002/wsbm.52 PMC3105527

[b14] Bachmann, . Predictive mathematical models of cancer signalling pathways. Journal of Internal Medicine 2, 155–165 (2012). 10.1111/j.1365-2796.2011.02492.x 22142263

[b15] Lee M. J. . Sequential application of anticancer drugs enhances cell death by rewiring apoptotic signaling networks. Cell 149, 780–794 (2012). 22579283 10.1016/j.cell.2012.03.031 PMC3501264

[b16] Saez-Rodriguez J. . Discrete logic modelling as a means to link protein signalling networks with functional analysis of mammalian signal transduction. Molecular Systems Biology 5, 331 (2009). 19953085 10.1038/msb.2009.87 PMC2824489

[b17] Schlatter R. . Modeling the TNF*α*-induced apoptosis pathway in hepatocytes. PloS One 6, e18646 (2011). 21533085 10.1371/journal.pone.0018646 PMC3080376

[b18] Sharan R. & Karp R. M. Reconstructing Boolean Models of Signaling. RECOMB 261–271 (2012). 10.1089/cmb.2012.0241 PMC3590894 23286509

[b19] Eduati F., De Las R. J., Di Camillo B., Toffolo G. & Saez-Rodriguez J. Integrating literature-constrained and data-driven inference of signalling networks. Bioinformatics (Oxford, England) 28, 2311–2317 (2012). 10.1093/bioinformatics/bts363 PMC3436796 22734019

[b20] Mishra S., Bhowmick S., Chua H., Zhang F. & Zheng J. Computational cell fate modelling for discovery of rewiring in apoptotic network for enhanced cancer drug sensitivity. BMC Systems Biology 9, S4 (2015). 10.1186/1752-0509-9-S1-S4 PMC4331679 25707537

[b21] Studham M., Tjarnberg A., Nordling T., Nelander S. & Sonnhammer E. Functional association networks as priors for gene regulatory network inference. Bioinformatics 30, i130–i138 (2014). 24931976 10.1093/bioinformatics/btu285 PMC4058914

[b22] Zhou F. & la Torre F. D. Canonical Time Warping for Alignment of Human Behavior. In Advances in Neural Information Processing Systems Conference (NIPS) 261–271 (2009).

[b23] Mukherjeea S. & Speed T. P. Network inference using informative priors. Proc Natl Acad Sci 105, 14313–14318 (2008). 18799736 10.1073/pnas.0802272105 PMC2567188

[b24] Prill R., Saez-Rodriguez J., Alexopoulos L., Sorger P. & Stolovitzky G. Crowdsourcing Network Inference: The DREAM Predictive Signaling Network Challenge. Sci Signal 4(189), mr7 (2011). 10.1126/scisignal.2002212 PMC3465072 21900204

[b25] Cao M. . New directions for diffusion-based network prediction of protein function: incorporating pathways with confidence. Bioinformatics 30, i219–i227 (2014). 24931987 10.1093/bioinformatics/btu263 PMC4058952

[b26] Morris M. K., Saez-Rodriguez J., Clarke D. C., Sorger P. K. & Lauffenburger D. A. Training signaling pathway maps to biochemical data with constrained fuzzy logic: quantitative analysis of liver cell responses to inflammatory stimuli. PLoS Computational Biology 7, e1001099 (2011). 21408212 10.1371/journal.pcbi.1001099 PMC3048376

[b27] Davey B. & Priestley H. Introduction to Lattices and Order (Cambridge University Press, 1990).

[b28] Cantone I. . A yeast synthetic network for *in vivo* assessment of reverse-engineering and modeling approaches. Cell 137, 172–181 (2009). 19327819 10.1016/j.cell.2009.01.055

[b29] Greenfield A., Hafemeister C. & Bonneau R. Robust data-driven incorporation of prior knowledge into the inference of dynamic regulatory networks. Bioinformatics 29, 1060–1067 (2013). 23525069 10.1093/bioinformatics/btt099 PMC3624811

[b30] Salvador S. & Chan P. FastDTW: Toward Accurate Dynamic Time Warping in Linear Time and Space. Intelligent Data Analysis 11, 561–580 (2007).

[b31] Mitsos A. . Non Linear Programming (NLP) formulation for quantitative modeling of protein signal transduction pathways. PloS One 7, e50085 (2012). 23226239 10.1371/journal.pone.0050085 PMC3511450

[b32] Mussel C., Hopfensitz M. & Kestler H. BoolNet–an R package for generation, reconstruction and analysis of Boolean networks. Bioinformatics 26, 1378–1380 (2010). 20378558 10.1093/bioinformatics/btq124

[b33] Tsamardinos I., Brown L. & Aliferis C. The max-min hill-climbing Bayesian network structure learning algorithm. Machine Learning 65(1), 31–78 (2006).

[b34] Aliferis C., Tsamardinos I. & Statnikov A. HITON, A Novel Markov Blanket Algorithm for Optimal Variable Selection. AMIA Annu Symp Proc 21–25 (2003). 14728126 PMC1480117

[b35] Aliferis C., Tsamardinos I. & Statnikov A. Causal Explorer: A Probabilistic Network Learning Toolkit for Biomedical Discovery. In METMBS ’03 (2003).

[b36] Zhang F. . Predicting Essential Genes and Synthetic Lethality via Influence Propagation in Signaling Pathways of Cancer Cell Fates. Journal of Bioinformatics and Computational Biology 13, 1541002 (2015). 25669329 10.1142/S0219720015410024

[b37] Zhang F. . Generalized logical model based on network topology to capture the dynamical trends of cellular signaling pathways. BMC System Biology 10(Suppl 1), 7 (2016). 10.1186/s12918-015-0249-9 PMC4895646 26818802

